# Supplementary figures and images for: Dacryocystitis: Is Dacryocystorhinostomy Always the Solution?
Source: J Clin Med. 2024 Aug 29;13(17):5129. doi: 10.3390/jcm13175129 (PMC11395886; doi:10.3390/jcm13175129)

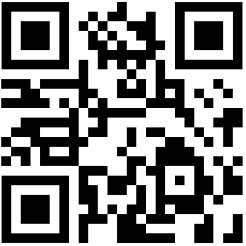

Supplement: Supplementary file 1 [file jcm-13-05129-s001.zip › jcm-3165310-supplementary.png]
